# Mobility Performance in Old Age: A Window Into Brain Integrity

**DOI:** 10.1093/geroni/igab046.613

**Published:** 2021-12-17

**Authors:** Qu Tian, Andrea Rosso, Caterina Rosano

## Abstract

Over two decades of research has established brain aging as a critical component of mobility decline. Studies consistently report that motor slowing predicts cognitive decline and neurodegenerative diseases, but reported associations are often modest. Both mobility and brain aging are complex processes and steady-state assessments are typically used (usual pace gait and structural MRI). We aim to elucidate the complex relations between brain aging and mobility by considering (a) strategies to maintain function such as interlacing periods of activity and rest (fractionation), (b) interventions that target brain and body (motor skill training), (c) multimodal neuroimaging (functional connectivity and cerebral small vessel disease (cSVD)), (d) challenged walking (dual-tasks, uneven surfaces), and (e) reduced resources (hearing loss). This symposium focuses on community-dwelling older adults from observational and intervention studies using state-of-the-art and real-life assessments of gait (quality and fragmentation by tri-axial accelerometry) and brain (near-infrared spectroscopy (fNIRS), resting-state functional MRI). First, we examine activity strategies that modify the relation between slow gait and AD risk (Tian). Second, using fNIRS, we investigate the extent to which motor skill training increases automaticity of gait (Chen). Third, we examine how functional connectivity may compensate for the detrimental effects of cSVD on mild parkinsonian signs (Hengenius). Fourth, we investigate the effects of challenged walking on gait quality and the relation with cognitive function (Suri). Finally, we demonstrate relations of hearing and cognition with mobility (Pupo). We seek to generate discussions on shared pathways underlying motor slowing and the aging brain and future prevention and intervention strategies.

